# Does the Prophylactic Power of Vaccination after a Certain Time Become Inoperative?

**Published:** 1854-01

**Authors:** Wm. G. Meacham

**Affiliations:** Dale, Wyoming Co., New York


ART. II.—*Does the Prophylactic Power of Vaccination after a certain time become inoperative?* By WM. G. MEACHAM, Dale, Wyoming Co., New York.

This is a query that has oftentimes presented itself to the inquiring medical mind, and one which has never irrefutably been answered. The amounts of argument adduced in support of the affirmative and the negative of this interrogatory which I have discovered in my examination of the subject thus far, are so evenly balanced, that were the question now put, and I required to give a positive, decisive "Aye," or "No," I should feel nearly as ill-prepared to open my lips as when I first began this interesting investigation. My labors, however, though they have not permanently, immovably settled the matter in controversy, though they have not conducted my mind into the clear, unclouded light of indisputable truth, though they have not removed every obstacle from the path to certainty, have yet dispelled many clouds from my mental canopy, have yet removed many impediments to my advance toward fixed, undoubted verity.

My principal object in now addressing this communication to you, Messrs. Editors, is to direct your valuable attention to the subject, and to call forth, if practicable and agreeable at the present time, your opinions and experience in relation to the matter *in lite;* as also to lay it before the numerous patrons and readers of your Journal, and thus possibly elicit their combined knowledge and experience.

The corner-stone argument upon which the advocates of the affirmative build their superstructure, seems to be this: The human physical system undergoes a complete change, a thorough renewal, in a period of about seven years' duration, as taught and believed by the profession generally. "Now," say they, "how can vaccinia perpetuate its protective, antivariolous efficacy in a bodily organization for a greater length of time than the actual, *identical* existence of that organization on which the disease exercised itself? The body, it is true, has the same form and symmetry at fifty years of age that it had at thirty, modified by time of life, accidents and other circumstances; but, *materially*, it is totally different, its substance, its particles are completely other. Now, can vaccination practiced on a certain aggregation of particles, continue its virtue longer than those particles remain in the man? Reason emphatically answers, 'No.'" I have here presumed the friends of the affirmative to be speaking personally—to be pleading their cause *propria persona.*

Let us see what can be offered in reply on the negative side. Even admitting the almost universally-accredited notion of the renovation of the system in a limited number of years, the foregoing argument is not unanswerable *in toto.* If the power of the cow-pox is exhausted in a certain time, by parity of reasoning, that of small-pox should be equally as well. If the one should fail, so should the other for precisely similar reasons. But, does observation prove this to be indeed the case? Does variola, or, more correctly speaking, its impression, leave the physical structure? Do we not well know that the truthful reply to this is, no? Are we not well convinced that this eruptive fever does not a second time invade the human frame? It is indeed asserted that a second and even a third attack of this scourge are occasionally endured by the same individual, but such cases, if any there are, are "like angels' visits, few and far between." In truth it is not impossible that those cases reported as second and third sieges of small-pox, may have been improperly diagnosticated, may have been confounded with one or other of those congenerous affections, varicella, rubeola, and scarlatina. But it is not essential here to question the assertion, for, if true, it does not militate against the *generalness* of freedom from more than one invasion of the *pustulous* monster; for what rule, not even barring the laws of nature, is without its exceptions? Now, if variola, scarlatina, and rubeola, exert an influence, make an impression on the corporal constitution, which are not limited to the self-same particles of matter on which that influence is primarily exerted, that impression first made, but which are transmitted to future fresh particles in the same individual, which are continued through his entire existence, if such be the case, I say, why should not their sister disease, vaccinia, prolong its efficacy through life?

Those who maintain that it is impossible, or at least improbable, that any disease can have an effect upon the human system for a greater length of time than the actual being of the matter composing that system at the period of the attack, seem to forget a plain, a universally-acknowledged law of our natures, the transmission of certain diatheses, peculiarities of constitution, normal or diseased conditions, &c., not only through the various stages of the person's life, but even to his descendants, his remote posterity. It is hardly necessary to direct attention to instances, for they are as familiar to the medical man as household words, and the school-boy even has often heard them referred to, and receives them as fixed facts with no less confidence and faith than he gives to the dicta of his master. The scrofulous or tuberculous cachexia and insanity are notable examples; also, as maintained by some, the arthritic fevers. In fact, for my own part, I see nothing so passing strange, so far beyond the reach of faith, in this doctrine of the continued protective agency of kine-pox during a lengthened period, it may be man's natural life, nor any incredibility in the hereditary opinion. To me they do not appear as "mystery, dumb amazement all."

The continued efficiency of vaccinia, if it exist, might be explained in this manner: When the disease has finished its course in the physical structure, it leaves an impression, sets its seal upon not only the material particles, but also the functions of the body, and particularly that of nutrition, so that the nutritive process is so modified that all future particles bear the same signet mark, antivariolous protection. We well know that a cicatrix is retained through life, as, for instance, that resulting from vaccination; why should not also the peculiar agency of vaccination in the system be preserved?

Again, the supporters of the affirmative of this question refer to the fact of the successful workings of a second, a third, a fourth, and other vaccinations at different periods in man's life, as evidence of the destruction of the vaccine virtue in a limited time. They say that if the effect of the original insertion of the virus were paramount, no succeeding insertions would be effectual. Is this a natural deduction, I will here inquire. May not a second, a third, or other operation *superadd* its effects to those of the former, which we may suppose to be still in activity? May not the army, vaccina, willingly receive reinforcements to assist it in resisting the powerful onslaughts of the *pitting* enemy, variola? But in reality the susceptibility of the system to the thorough impression of cow-pox is never as great after as before the first attack. Probably no larger proportion than one-half of the revaccinations made, ever induce a well-formed course of vaccinia. So far as my individual experience extends, I can, I *must* say that revaccination is very unsatisfactory. Upon not one of thirty or more previously vaccinated subjects upon whom I recently operated, did the virus produce any reliable effects. In some of the cases slight redness and swelling around the punctures were perceptible for a few days, and then died away. In others, no sensible results, local or constitutional, followed. It is, indeed, possible that the virus may not have been of the best quality; but I have reason to credit its virtue, for it operated successfully on several *unprotected* subjects, thus demonstrating that it had efficacy on the unvaccinated, and none on those who had previously undergone an assault of kine-pox. This isolated, this sporadic (pardon this anomalous use of the word, for medical terms are uppermost in my mind) experience does not, of course, prove or disprove the doctrine of permanency of the prophylactic power of vaccination; but it is not utterly valueless, for all experience is but the aggregation of individual experiences. The failures above mentioned are not due to faulty or imperfect insertion, but are attributable either to deficient energy in the virus, or (which is the most probable) to the still active efficacy of the former vaccination. That they are not due to defective strength in the virus seems demonstrable by the fact, as before stated, of the successful insertion of the same virus in several unprotected persons. We therefore seem necessitated to attribute them to the still operative force of prior vaccinia, as a *dernier resort*. It is quite evident that these thirty persons did not possess constitutions unsusceptible to the peculiar agency of cow-pox, for they had all had vaccinia at an antecedent date; and it is quite improbable that the virus made its exit from the punctures in all these thirty cases before it had been absorbed. I therefore seem instinctively impelled to take hold of the still efficient virtue of the first cow-pox as a solution of the mystery, somewhat as a drowning man catches at a straw.

The conclusion to which my investigation of this subject has led me is this: The effect produced by vaccination on the system is not limited in duration to the continuance in the body of the identical particles on which the effect was primarily made, but extends down to a more remote period, perhaps through life. I do not maintain, however, that its influence endures *unimpaired* through all this time. It doubtless gradually diminishes in activity, and perchance eventually forsakes the body, or at least is so far gone as to be of little utility. Hence, it seems the part of wisdom to have recourse again and again to vaccinia antivariolosa, for it cannot, as I conceive, possibly do harm, and may result in great, invaluable good.

But, whether the protective virtue of vaccinia is transient or permanent, whether it continues for seven years, or for that number doubled, or trebled, or quadrupled, or for life, what praises are due unto thy great name, immortal Jenner, for the inestimable blessings which thou hast conferred upon mankind by introducing into general use the practice of vaccination, by which that pustulous monster which once so terribly ravaged the earth, has been shorn of his strength, by which his power has been crushed, and his fury checked! The gratitude and adoration of unnumbered millions, and the approval of Him whose favor is better than life, and whose frown is destruction, are thy rich reward!

